# Pharmacokinetic analysis of nacubactam, a novel β-lactamase inhibitor, co-administered with meropenem in patients with a complicated urinary tract infection

**DOI:** 10.1093/jacamr/dlag098

**Published:** 2026-06-03

**Authors:** Jun Morita, Kazuya Ishiwata, Shogo Matsumoto, Seiji Kato, Gemma Attley, Katie Patel, Ignacio Rodriguez, Navita Mallalieu

**Affiliations:** R & D Division, Meiji Seika Pharma Co., Ltd., Tokyo, Japan; R & D Division, Meiji Seika Pharma Co., Ltd., Tokyo, Japan; R & D Division, Meiji Seika Pharma Co., Ltd., Tokyo, Japan; R & D Division, Meiji Seika Pharma Co., Ltd., Tokyo, Japan; Roche Pharma Research and Early Development, Roche Innovation Centre New York, Little Falls, NJ, USA; Global Product Development, Roche Products Limited, Welwyn Garden City, UK; Roche Pharma Research and Early Development, Roche Innovation Centre New York, Little Falls, NJ, USA; Roche Pharma Research and Early Development, Roche Innovation Centre, New York, NY, USA

## Abstract

**Objectives:**

Nacubactam is a novel non-β-lactam β-lactamase inhibitor being developed to treat serious Gram-negative bacterial infections. We report data from the first clinical study evaluating nacubactam intravenous (more than 1.5 h) administered to adults with presumed/confirmed complicated urinary tract infections (cUTIs), including pyelonephritis.

**Patients and methods:**

In this non-randomized, open-label study, 20 patients (mean age 51.5 years; 85.0% white; *n* = 13 females and *n* = 7 males) received intravenous infusions (more than 1.5 h) of 2 g nacubactam co-administered with 2 g meropenem three times daily (every 8 h) for 3–14 days.

**Results:**

Nacubactam pharmacokinetic (PK) data were similar to those obtained in prior volunteer studies. With one exception (severe nausea), adverse events (AEs) were of mild to moderate intensity, with no new safety signals, severe AEs or death. Fifty per cent had achieved joint microbiological eradication and clinical cure. Exploratory efficacy analyses in eight evaluable patients with confirmed Gram (−) cUTI identified that at follow-up (ITT population), 75.0% had achieved a clinical response of cure and 62.5% had achieved a microbiological response of eradication. Predicted PK/PD indices suggested that all 20 patients would achieve exposure targets for meropenem (minimum inhibitory concentration of 4 mg/L) and nacubactam (8 mg/L).

**Conclusions:**

The consistent PK and safety profiles of nacubactam, and the ability to align the dosing regimen with partner antibiotics, confirm the suitability of nacubactam for further clinical development in patients with Gram-negative infections.

## Introduction

Antimicrobial resistance (AMR) means that research into new antibiotics to mitigate infection-related mortality over the coming decades is a critical clinical concern.^1^ There are particularly high rates of AMR among the Gram-negative uropathogens which cause complicated urinary tract infections (cUTIs),^2^ stimulating intensive research into novel therapeutic and preventive strategies.^3^

The novel non-β-lactam β-lactamase inhibitor nacubactam (OP0595, RO7079901) is a member of the diazabicyclooctane (DBO) class of compounds and is in clinical development for the treatment of serious Gram-negative bacterial infections.^4,5^ Nacubactam has a dual mechanism of action (MOA), which involves both direct antibacterial effects via inhibition of penicillin-binding protein 2 (PBP-2) in Enterobacteriaceae, plus indirect effects via inhibition of serine β-lactamases, thereby protecting partner β-lactam antibiotics from degradation.^6,7^ In a murine model of cUTI, nacubactam combined with the β-lactam antibiotic meropenem was shown to enhance the activity of either agent alone, particularly against meropenem-resistant Enterobacteriaceae isolates, and also increased antibacterial kill against ceftazidime-avibactam-resistant isolates.^8^

Phase 1 clinical studies in human volunteers have provided initial pharmacokinetic (PK) and safety data for single and multiple intravenous (IV) doses of nacubactam, alone or in combination with meropenem.^4^ In this non-randomized, open-label study, we evaluated the PK and safety of nacubactam IV administered (more than 1.5 h) three times daily (every 8 h), in combination with meropenem, in a population of male and female adult patients with a cUTI caused by a Gram-negative bacterium.

## Materials and methods

### Study design

This was a non-randomized, open-label, one treatment, one group study conducted in adults with a cUTI (including pyelonephritis). The study was conducted at 10 investigational sites in four countries (three sites in Latvia, three sites in Serbia, two sites in Poland and two sites in the USA).

The study consisted of a screening visit, a dosing period and a safety follow-up period. During screening, blood and urine samples were collected to confirm eligibility. The duration of study drug treatment was determined by the investigator based on their medical judgment after evaluation of the patient’s response. Each enrolled patient received IV infusions of 2 g nacubactam co-administered with 2 g meropenem (more than 1.5 h) every 8 h, for a minimum of 3 days (i.e. 9 infusions) and a maximum of 14 days (i.e. 42 infusions). Nacubactam and meropenem were administered concurrently. If the investigator chose to discontinue study treatment, they could select an oral antibiotic or a different IV agent to complete a minimum of 7 days total therapy. Patients returned for two follow-up visits, 1 and 2 weeks after completing study treatment (i.e. between Days 10–21 and Days 17–28).

### Study population

Eligibility required clinical signs/symptoms suggestive of pyelonephritis, specifically at least two of the following: fever associated with chills, rigours or sensation of warmth; flank pain or pelvic pain; costovertebral angle pain or suprapubic tenderness; nausea/vomiting; and dysuria, frequency or urgency. In addition, a urine specimen with evidence of pyuria was necessary: either a dipstick analysis positive for leukocyte esterase or ≥1 × 10^5^ white blood cells (WBCs)/mL (corresponding to 10 WBCs/mm^3^) or >5 WBCs/high power field. Patients were also required to have a urine culture taken within the 48-h period immediately preceding the first dose of study drug containing >1 × 10^5 ^cfu/mL of a Gram-negative bacterium and a negative pregnancy test or agreement to remain abstinent/use contraception during treatment. Despite microbiological entry criteria of >10^5 ^cfu/mL Gram-negative bacteriuria, eight patients met this threshold. The primary analysis included clinically confirmed cases regardless of exact titre, per standard cUTI practice. All eligible patients provided written informed consent for study participation.

Key exclusion criteria were concomitant infection requiring another antibacterial therapy; confirmed fungal UTI; moderate or severe renal impairment, requirement for renal replacement therapy or recipient of a renal transplant; documented presence of immunodeficiency, or a severely immunocompromised condition or use of systemic immunosuppressant therapy; any rapidly progressing, life-threatening or terminal condition; urogenital surgery or trauma within 1 week prior to study start; UTI symptoms attributable to another cause; suspected or confirmed perinephric or intra-renal abscess; urinary obstruction or diversion; use of probenecid within one week prior to study start; hypersensitivity to antibiotics; or any other condition rendering the patient unsuitable for study enrolment. No restrictions were placed on prior antibiotic use or duration. The study was conducted in accordance with the principles of the Declaration of Helsinki and Good Clinical Practice.

Exploratory efficacy objectives were to explore microbiological and clinical responses to treatment, to explore the relationship between exposure to study treatment and microbiological and clinical responses and to evaluate *in vitro* sensitivity of causative uropathogens to the nacubactam/meropenem treatment combination. Microbiological eradication required that the cUTI causative pathogen in urine was reduced to <10^4 ^cfu/mL. A patient was considered clinically improved if afebrile (<38°C), haemodynamically stable and demonstrating improved signs and symptoms of cUTI or pyelonephritis. The Patient Symptom Assessment Questionnaire was also used to elicit feedback on overall symptom relief (recorded as no, mild, moderate or severe symptoms) in order to evaluate response. All enrolled patients were scheduled to undergo intensive PK blood sampling on Days 1 and 3, with additional sparse sampling on Days 5 and 7. The sample size was determined based on feasibility, and no statistical power calculations were conducted. It was planned to enrol 20 evaluable patients (i.e. patients who received at least nine infusions of study treatment), including at least five patients with acute pyelonephritis and a fever, if possible.

All patients who received at least one dose of the study medication and had data from at least one post-dose PK sample were included in the PK analysis population. All patients who received at least one dose of the study medication and who had ≥1 × 10^5 ^cfu/mL of a recognized uropathogen from the baseline urine culture were included in the mITT population for exploratory efficacy analyses. All patients who received at least one dose of the study medication were included in the safety analysis population.

PK parameters for nacubactam and meropenem were derived from plasma and urine concentration data according to standard non-compartmental analysis methods using Phoenix WinNonlin version 6.4 (Certara Inc., Princeton NJ, USA). Relative concentrations of meropenem and nacubactam at each nominal sampling time point were derived from plasma concentrations of the two analytes (expressed as a decimal fraction, without adjustment for molecular weight).

Exploratory efficacy analyses were summarized descriptively by time point. Safety data were also summarized descriptively. Adverse events (AEs) were categorized using Preferred Terms from the Medical Dictionary for Regulatory Activities version 20.0. Statistical analyses were conducted using SAS software version 9.4 (SAS Institute, Inc., Cary, NC, USA).

### Trial registration

The study described in this paper was registered at ClinicalTrials.gov with the identifier NCT03174795.

## Results

### Study population

Of 24 patients who were screened for eligibility, 20 (*n* = 13 females and *n* = 7 males) were enrolled, and 19 completed the study. In addition to the patient who was withdrawn early, two additional patients discontinued treatment early [one due to AEs (severe, mild and moderate)] and one due to a positive yeast culture indicating that the UTI was not due to a Gram (−) bacterium.

All 20 enrolled patients who received 2 g nacubactam and 2 g meropenem IV infusion (more than 1.5 h) every 8 h had nacubactam and meropenem plasma and urine concentration data available for subsequent PK analysis. The median duration of exposure was 5.5 days (range: 3–13 days). The median total cumulative dose of nacubactam and meropenem was 27 g (nacubactam range: 14–71 g; meropenem range: 14–72 g).

Baseline characteristics are shown in Table 1. The mean age was 51.5 years, most patients were white [17/20 (85.0%)], and almost two-thirds were female [13/20 (65.0%)]. Creatinine clearance (CrCl) tended to be lower among patients older than 60 years of age (Figure S1, available as Supplementary data at *JAC-AMR* Online). Overall, the baseline demography and anthropometric characteristics were consistent with expectations for a population of patients with a cUTI.

**Table 1.** Summary of demographic and baseline characteristics

	Nacubactam/meropenem (*n* = 20)
Age, years	
Mean (SD)	51.5 (18.4)
Median (range)	50.0 (18–86)
<65, *n* (%)	14 (70.0)
Female sex, *n* (%)	13 (65.0)
Race, *n* (%)	
White	17 (85.0)
African-American	1 (5.0)
Unknown	2 (10.0)
Ethnicity, *n* (%)	
Not Hispanic or Latino	15 (75.0)
Hispanic or Latino	3 (15.0)
Not stated or unknown	2 (10.0)
BMI, kg/m^2^	
Mean (SD)	27.6 (4.9)
Median (range)	27.3 (18.7–35.5)
Creatinine clearance, mL/min^a^	
Mean (range)	107 (36–158)

SD, standard deviation.

^a^Derived from serum creatinine using the method of Cockroft and Gault.

Eight patients who received at least one dose of the study medication and who had ≥1 × 10^5^ colony forming units (cfu)/mL of a recognized uropathogen from the baseline urine culture were included in the microbiologic intention-to-treat (mITT) population for exploratory efficacy analyses.

### Nacubactam PK

**Figure 1.**
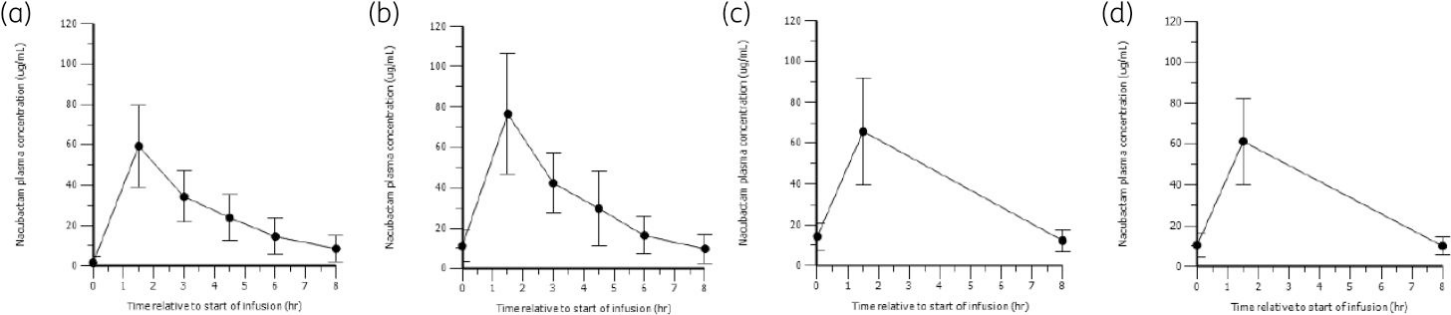
Mean (±SD) plasma concentration-time profiles of nacubactam after dosing with 2 g nacubactam co-administered with 2 g meropenem every 8 h. (a) Day 1 (*n* = 20); (b) Day 3 (*n* = 19); (c) Day 5 (*n* = 10); (d) Day 7 (*n* = 6). SD, standard deviation; every 8 h, three times daily.

Mean nacubactam plasma concentration s are shown in Figure 1. Peak nacubactam plasma concentrations occurred at the end (more than 1.5 h) of the IV infusion, and thereafter, plasma concentrations declined in an apparently biphasic manner. Nacubactam PK did not change with repeat dosing: plasma concentration versus time profiles on Day 3 were similar to those on Day 1, and profiles from sparse sampling on Days 5 and 7 among the subset of patients who continued treatment were also consistent. The study samples were stored at −80°C and no stabilizers such as MOPS were used; only physiological saline solution was used. The BLQ information is 1 µg/mL.

Corresponding derived nacubactam PK parameters for Days 1 and 3 are summarized in Table 2. There was no apparent change in plasma CL, volume of distribution at steady state (V_ss_) or CL_r_ between Days 1 and 3. Plasma exposures were higher on Day 3 than Day 1 in some individuals [particularly those with lower renal function (male eGFR < 50)]; consequently, average peak and total exposures were also marginally higher on Day 3. Variability in all plasma PK parameters was moderate (e.g. 49% for CL and 37% for V_ss_ on Day 1) and did not change with repeat dosing. Approximately 60% (Day 1 and Day 3) of the total administered nacubactam dose was recovered unchanged in the urine during the 8-h urine collection period. Estimates of renal clearance were below corresponding estimates of total clearance and also exhibited high inter-individual variability.

**Table 2.** Summary of PK parameters^a^ after dosing of 2 g nacubactam co-administered with 2 g meropenem every 8 h

Parameter	Nacubactam		Meropenem	
	Day 1 (*n* = 20)	Day 3 (*n* = 19)	Day 1 (*n* = 20)	Day 3 (*n* = 19)
CL, L/h	8.83 (49)	8.05 (46)	19.4 (63)	17.2 (52)
V_ss_, L	31.3 (39)	26.0 (41)	46.3 (64)	34.4 (61)
AUC_0–8h,_ µg.h/mL	195 (42)	248 (46)	97.6 (59)	116 (52)
AUC_0-inf,_ µg.h/mL	227 (49)	ND	103 (63)	ND
C_max_, µg/mL	54.4 (38)	70.9 (45)	34.3 (59)	44.5 (54)
t_max_, h	1.50 (1.5–3.6)	1.75 (1.5–4.5)	1.50 (1.5–3.6)	1.50 (1.5–3.1)
t_1/2_, h	2.26 (39)	2.18 (34)	1.42 (38)	1.28 (25)
A_e_ (mg)	1130 (96)^b^	1180 (67)^c^	978 (79)^b^	1041 (49)^c^
f_e_ (%)	56.4 (96)^b^	59.3 (67)^c^	48.9 (79)^b^	52.0 (49)^c^
CL_r_ (L/h)	5.84 (94)^b^	4.66 (85)^c^	10.3 (98)^b^	8.88 (89)^c^

Data are shown as geometric mean (coefficient of variation %), except t_max_ which is shown as median (range).

A_e_, cumulative amount excreted in urine from time zero to 8 h; AUC_0–8h_, area under the concentration-time curve from 0 to 8 h; AUC_0-inf_, area under the concentration-time curve from 0 h to infinity; CL, total plasma clearance; CL_r_, renal clearance; C_max_, maximum observed concentration; f_e_, fraction of administered drug dose excreted in urine from time zero to 8 h; ND, not determined; PK, pharmacokinetics; t_1/2_, terminal half-life; every 8 h, three times daily; t_max_, time of maximum plasma concentration (relative to start of infusion); Vss, volume of distribution at steady state.

^a^The protocol did not stipulate which of the three scheduled daily study drug dosing events was to be used as the reference event for PK sampling, and choice of reference dosing event on each PK assessment day was at the discretion of the investigator. Thus, study drug concentrations in predose samples on Day 1 were non-zero in some individuals because PK blood sampling was linked to the second or third study drug doses administered.

^b^ *n* = 19.

^c^ *n* = 18.

The influence of demographic and baseline parameters on nacubactam PK was explored visually, using graphical presentations of primary and secondary PK parameters. The only covariate which appeared to have an impact was CrCl, which affected exposure and total plasma clearance.

### Meropenem PK

**Figure 2.**
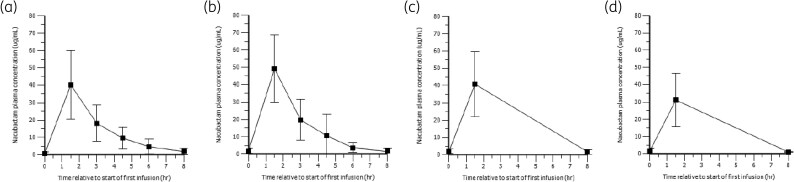
Mean (±SD) plasma concentration-time profiles of meropenem after dosing with 2 g nacubactam co-administered with 2 g meropenem every 8 h. (a) Day 1 (*n* = 20); (b) Day 3 (*n* = 19); (c) Day 5 (*n* = 10); (d) Day 7 (*n* = 6). SD, standard deviation; every 8 h, three times daily.

Mean meropenem plasma concentration versus time profiles are shown in Figure 2. Results were similar to those observed with nacubactam: peak meropenem plasma concentrations occurred at the end of the IV infusion (more than 1.5 h), followed by biphasic decline, and there was no change in PK with repeat dosing.

Corresponding derived meropenem PK parameters for Days 1 and 3 are summarized in Table 2. There were minimal changes in primary and secondary PK parameters, or variability, between Days 1 and 3. Approximately half of the administered meropenem dose was recovered unchanged in the urine during the 8-h urine collection period. The study samples were stored at −80°C and no stabilizers such as MOPS were used; only physiological saline solution was used. The BLQ information is 0.1 µg/mL.

### Meropenem:nacubactam relative concentrations

Plasma concentrations of the two agents were initially similar (decimal fraction ∼0.7); meropenem concentrations were markedly lower than corresponding nacubactam concentrations at the end of the dosing interval (decimal fraction < 0.2). Despite these PK differences, both drugs were maintained.

### Exploratory efficacy analyses

Overall severity of cUTI symptoms was moderate to severe in most patients at baseline and improved to no symptom or mild in all patients by the end of treatment.

By the follow-up period, 6/8 patients (75.0%) had achieved a clinical response of cure (i.e. complete resolution of the signs and symptoms of cUTI, with no further antimicrobial therapy required), 5/8 patients (62.5%) had achieved a microbiological response of eradication (i.e. cUTI causative pathogen in urine was reduced to <10^4 ^cfu/mL), and 4/8 patients (50.0%) had achieved joint microbiological eradication and clinical cure.

Minimum inhibitory concentrations (MICs) for nacubactam, meropenem and meropenem plus nacubactam at baseline for the 13 patients with Gram-negative bacterial uropathogens are summarized in Table S1. Patients without results had no culturable Gram-negative bacterial uropathogen. Based on published breakpoints^9,10^ for meropenem at the time of this analysis (1 and 2 mg/L for the approved 1 g every 8 h dose administered over 30 min), all isolated pathogens were susceptible to meropenem monotherapy (2 g every 8 h). Plots of individual % time > target concentration values versus corresponding target concentrations are shown in Figure S1.

### Safety

A total of 20 AEs were reported in 8 (*n*: 1, male; *n*: 7, female)/20 patients (40.0%). All were of mild to moderate intensity, with the exception of one event of severe nausea. There were no deaths. The patient who experienced severe nausea also experienced moderate diarrhoea and discontinued study treatment. One patient (5.0%) experienced a serious AE of moderate musculoskeletal chest pain that resulted in interruption of nacubactam and meropenem treatment, but was considered unrelated to study treatment.

The only AEs occurring in more than one patient were nausea [3/20 (15.0%)] and vomiting [2/20 (10.0%)]. Two AEs were considered related to both nacubactam and meropenem (diarrhoea and genital fungal infection), one AE was considered related to nacubactam alone (hypersensitivity), and five were considered related to meropenem alone (nausea, *n* = 3; vomiting, *n* = 2). [There is no evidence that nacubactam can cause genital fungal infection while it has been widely demonstrated that MEROP can cause GFI (vaginal yeast infections mainly!). MEROP is known to cause secondary fungal infections/fungal overgrowth.] One patient was discovered to be pregnant during the study after receiving nine doses of study medication. As of the final visit, the pregnancy was progressing as expected. There were no clinically relevant abnormalities in laboratory test results, ECG parameters, or vital signs, or any related AEs reported during the study.

## Discussion

In this first clinical study evaluating nacubactam IV administered every 8 h, for a minimum of 3 days (and a maximum of 14 days with a confirmed cUTI, we investigated the PK of nacubactam and found results were similar to those obtained in Phase I studies in healthy volunteers and in volunteers with moderate, mild or severe renal impairment.^4^ Given the ∼30% variability of what in healthy volunteers and the fact that antibiotic distribution and clearance can be different in patient populations,^11^ the differences observed between studies were not considered to indicate fundamental differences in nacubactam disposition in patients with cUTI. Meropenem plasma exposures and cumulative urinary excretion results were generally consistent with those reported.^12,13^

Respective maximum and total exposures for nacubactam in this study following a 2 g dose co-administered IV infusion (more than 1.5 h) every 8 h for 3 to 14 days with 2 g meropenem were 54 µg/mL and 227 µg.h/mL, which is similar to the respective exposures of 65 µg/mL and 224 µg.h/mL previously reported in healthy volunteers. Average estimated CL (8.83 L/h) and V_ss_ (31.3 L) in this patient population were also similar to those in healthy volunteers (8.93 L/h and 25.8 L, respectively).

Approximately 60% of the administered nacubactam dose was recovered unchanged in the urine during the 8-h urine collection period, although considerable inter-individual variability was observed. A similar pattern of highly variable results was observed for meropenem, and there were no obvious protocol deviations noted that would explain the high variability, which suggested the cause may be practical difficulties in accurately collecting and pooling urine samples in a multicentre patient study. Nonetheless, the results support previous results^4^ indicating that a large fraction of the administered nacubactam is eliminated unchanged via renal excretion. There was an apparent direct relationship between renal function and nacubactam clearance, which was consistent with previous observations, meaning that nacubactam dosing recommendations will need to account for the level of residual renal function to ensure that exposures are maintained within an appropriate range.

The ability to administer IV infusion every 8 h for 3 to 14 days nacubactam every 8 h offers the potential to align the dosing regimen with other antibiotics, such as meropenem, aztreonam and cefepime, all of which have been proposed as partners for nacubactam for clinical use. In addition, the use of a 1.5-h infusion for meropenem may have limited the duration of target concentration maintenance, particularly in patients with higher drug clearance. Longer or extended infusions of meropenem have been shown to enhance %T > MIC for β-lactam antibiotics, and optimization of infusion duration may warrant consideration in future clinical studies evaluating nacubactam combinations.

Despite favourable predicted PK/PD target attainment based on low baseline MIC values, the observed clinical response rate of 75% appeared lower than might be expected for meropenem-susceptible pathogens. This discrepancy highlights the inherent limitations of extrapolating clinical outcomes from PK/PD indices alone, particularly in a small, heterogeneous patient population. Several factors may have contributed to this finding, including variability in disease severity, differences in host response, prior antibiotic exposure before enrolment and the limited duration of IV therapy. In addition, microbiological confirmation meeting strict entry criteria was available in only a subset of patients, which further constrains interpretation of efficacy outcomes. The meropenem dosing regimen employed used a 1.5-h IV infusion administered every 8 h, which reflects a pragmatic balance between clinical feasibility and enhanced exposure compared with standard short infusions.

This study has several limitations inherent to its Phase 2 exploratory design. The small sample size, open-label structure, absence of a comparator arm and variability in treatment duration and post-treatment antibiotic management limit the generalizability of both PK and clinical outcome findings. Furthermore, the primary objective of this study was characterization of PKs rather than formal assessment of efficacy. As such, efficacy-related observations should be interpreted cautiously and were included to provide supportive context rather than definitive conclusions. Future studies with larger sample sizes and standardized treatment protocols will be required to more robustly define the clinical impact of nacubactam combination therapy.

Our data showed that in the mITT population, 50% of patients achieved a joint microbiological eradication and clinical cure at the first follow-up visit (Days 10–21) although separately 75% achieved a clinical response of cure, and 62.5% had a microbiological response of eradication. However, only 13 patients had Gram-negative bacterial uropathogens culturable from baseline samples sent to the central susceptibility testing laboratory, even though all 20 patients met the study entry criterion requiring evidence of a Gram-negative bacterial infection. The inconsistency is likely attributable to the fact that entry was based on suspected infection (prior to receipt of culture results due to turnaround time), and patients that received empirical treatment upon admission (prior to screening) were not excluded. In addition, even though MIC data from *in vitro* susceptibility tests indicated all causative Gram-negative pathogens were susceptible to meropenem,^9,10^ while enrolment of patients with prior antibiotic exposure, variable treatment durations (median 5 days), lack of standardized oral step-down protocols the favourable outcomes with only 5 days of therapy align with antimicrobial stewardship principles favouring shorter treatment courses. No restrictions were placed on prior antibiotic use or duration.

Given the low MIC values observed in this study, all pathogens were susceptible to both meropenem and the combination, achieving the presumed PK/PD targets. Instead, theoretical PK/PD indices were calculated for all 20 patients using observed nacubactam concentrations to predict PK/PD index values for a range of different target concentrations. β-lactam antibiotics such as meropenem exhibit primary time-dependent antibiotic effects, and percentage time over a target MIC (%T > MIC) of 40% is widely accepted as an appropriate PK/PD index target for meropenem.^14^ Similarly, microbiology data from *in vitro* studies and *in vivo* animal models demonstrated that the meropenem/nacubactam combination exhibited time-dependent effects, with %T > MIC being the most relevant PK/PD index for efficacy and a target of 42%.^15–17^ Using these values, it is expected that all 20 patients would achieve meropenem exposure targets for an MIC of 4 mg/L and nacubactam exposure targets for an MIC of 8 mg/L on both Days 1 and 3. The recently completed Phase III Integral-1 study is expected to provide detailed efficacy data on nacubactam antibiotic combinations other than meropenem in patients with cUTI, with initial reports indicating that the trial met the primary endpoint of composite clinical and microbiological success at 7 days after the end of treatment.^18^

Safety analyses in this study indicated that IV administration (more than 1.5 h) of 2 g of nacubactam together with 2 g meropenem every 8 h for up to 14 days was well tolerated by patients with cUTI. No unexpected or clinically relevant safety signals (severe AEs) were identified, and the reported AEs were in line with the published safety profiles of the study treatments.^4,12,13,19^

In conclusion, the results from this study have quantified nacubactam PK parameters in patients with cUTIs, and confirmed a consistent PK and safety profile suitable for further clinical investigation in patients with Gram-negative infections. The critical need for new treatments for Gram-negative bacteria, to overcome current therapeutic challenges related to resistance, means that there is great interest in novel β-lactam/β-lactamase inhibitor combinations. As such, efficacy data from larger-scale clinical studies using nacubactam combination treatments are eagerly anticipated.

## Supplementary Material

dlag098_Supplementary_Data

## Supplementary data

Figure S1 and Table S1 are available as Supplementary data at *JAC-AMR* Online.
