# Supplementary material for: Pharmacokinetic analysis of nacubactam, a novel β-lactamase inhibitor, co-administered with meropenem in patients with a complicated urinary tract infection
Source: JAC Antimicrob Resist. 2026 Jun 3;8(3):dlag098. doi: 10.1093/jacamr/dlag098 (PMC13231170; doi:10.1093/jacamr/dlag098)
Supplement: dlag098_Supplementary_Data [file dlag098_supplementary_data.docx]

**Supplementary data**

SUPPLEMENTARY TABLE 1 Baseline minimum inhibitory concentrations for causative Gram-negative uropathogens

| Pathogen | Meropenem | Nacubactam | Meropenem + nacubactam | |
| --- | --- | --- | --- | --- |
|  |  |  | 1:1 fixed ratio | 4 µg/mL fixed concentration |
| Escherichia coli | 0.015 | 1 | 0.015 | ≤0.004 |
| Escherichia coli | 0.015 | 1 | 0.015 | ≤0.004 |
| Escherichia coli | 0.015 | 1 | 0.03 | ≤ 0.004 |
| Escherichia coli | 0.015 | 2 | 0.015 | ≤0.004 |
| Escherichia coli | 0.03 | 2 | 0.03 | ≤0.004 |
| Escherichia coli | 0.03 | 2 | 0.03 | ≤0.004 |
| Escherichia coli | 0.03 | 4 | 0.015 | ≤0.004 |
| Klebsiella pneumoniae | 0.03 | 4 | 0.03 | ≤0.004 |
| Klebsiella pneumoniae | 0.03 | 256 | 0.03 | ≤0.004 |
| Klebsiella pneumoniae | 0.03 | > 256 | 0.03 | 0.008 |
| Klebsiella pneumoniae | 0.03 | >256 | 0.03 | 0.015 |
| Klebsiella pneumoniae | 0.06 | 4 | 0.06 | 0.008 |
| Proteus mirabilis | 0.12 | 4 | 0.03 | ≤0.004 |

SUPPLEMENTARY FIG. 1 Plots of percentage time observed study drug concentrations exceeded theoretical target concentrations. (A) Nacubactam Day 1; (B) nacubactam Day 3; (C) meropenem Day 1; (D) meropenem Day 3

A


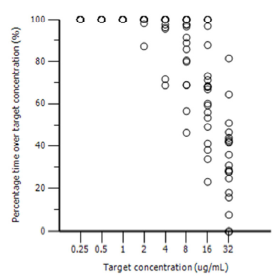


B


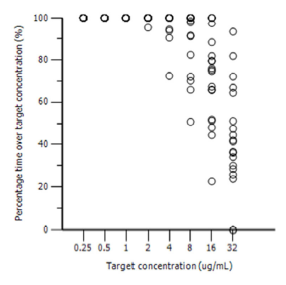


C


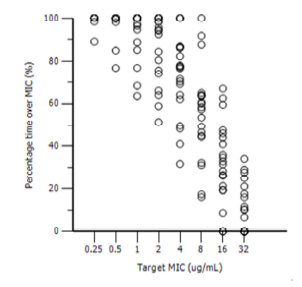


D


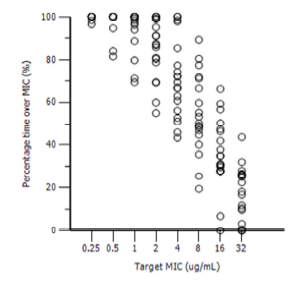


MIC, minimum inhibitory concentration.
